# The clinical effects of Orff music therapy on children with autism spectrum disorder: a comprehensive evaluation

**DOI:** 10.3389/fneur.2024.1387060

**Published:** 2024-06-06

**Authors:** Qiongli Fan, Mengying Ding, Wang Cheng, LiSha Su, Yuping Zhang, Quanxing Liu, Zhifeng Wu

**Affiliations:** ^1^Department of Pediatrics, Second Affiliated Hospital of Army Medical University, Chongqing, China; ^2^Department of Pediatrics, The General Hospital of Western Theater Command, Chengdu, China; ^3^Department of Thoracic Surgery, Second Affiliated Hospital of Army Medical University, Chongqing, China

**Keywords:** autism spectrum disorder, Orff music therapy, comprehensive rehabilitation intervention, children, ABC, CARS, PEP-3

## Abstract

**Objective:**

This study aimed to investigate the clinical effects of Orff music therapy on children with Autism Spectrum Disorder (ASD) from the perspectives of parents, evaluators, and therapists.

**Methods:**

93 children with ASD aged 3–6 years participated in the study. They were divided into an observation group (*n* = 48) receiving comprehensive rehabilitation intervention including Orff music therapy, and a control group (*n* = 45) receiving only comprehensive rehabilitation intervention. The Autism Behavior Checklist (ABC), Childhood Autism Rating Scale (CARS), and Psycho-educational Profile-3rd edition (PEP-3) were used for assessments before and after the intervention.

**Results:**

There were no significant demographic differences between the two groups. Both groups showed significant improvements in Sensory, Relating, Language, CVP, EL, RL, VMI, AE, SR, and CARS scores at T1, T2, and T3 (T1 vs. T2, T2 vs. T3, T1 vs. T3) (all *p* < 0.05). The observation group demonstrated significant changes in Body and Object use and FM, while the control group showed some changes in these domains. Social and self-help, GM, CMB, and CVB also significantly improved in both groups after 6 months of intervention (all *p* < 0.05). In terms of different time intervals, the observation group showed greater improvements in Sensory, Relating, Language, CARS scores, EL, RL, and SR compared to the control group (all *p* < 0.05). The improvement levels in Body and Object use, CVP, FM, VMI, and AE did not differ significantly between the two groups in the T1–T2 interval, but were significantly higher in the observation group in the T2–T3 and T1–T3 intervals (all *p* < 0.05). The magnitude of changes in Social and self-help, GM, CMB, and CVB did not differ significantly between the groups.

**Conclusion:**

Orff music therapy showed significant improvements in language expression, language comprehension, social skills, cognitive abilities, imitation abilities, emotional expression and fine motor in children with ASD. These findings provide support for the use of Orff music therapy as an effective intervention for children with ASD.

## 1 Introduction

Autism Spectrum Disorder (ASD) is a severe neurodevelopmental disorder that typically appears in early childhood and is characterized by social communication and interaction difficulties, as well as repetitive and restrictive behaviors (1). According to the Centers for Disease Control and Prevention (CDC) in the United States, the prevalence of ASD has increased from 2 in 10,000 to 1 in 54 over the past 20 years (2). The global prevalence of ASD is estimated to be 1–2% (3). A multicenter study conducted by Sun et al. in China in 2019 reported a prevalence of approximately 1% for children with ASD in China (4). In comparison with children with normal development, children with ASD may experience social difficulties in early stages, which may result in significant deviations in their later development, such as delayed achievement of developmental milestones in establishing peer and family relationships. In addition, social impairment may also have a negative impact on their social and vocational functioning in adulthood, while also placing a significant economic burden on their families and society (5, 6). Currently, there are no pharmacological treatments available for the core symptoms of ASD, and without effective interventions, 60–80% of individuals with ASD will experience lifelong disability (7). However, research has shown that early detection and intervention can significantly improve the unfavorable prognosis of children with ASD (8). Currently, there are many effective methods for intervention in ASD, primarily focusing on early education and behavioral correction. The aim of intervention is to improve core symptoms, promote the development of social interaction abilities, speech and non-verbal communication abilities, reduce repetitive behaviors, promote intellectual development, foster adaptive and independent living skills, reduce maladaptive behavior, alleviate the degree of disability, improve quality of life, and reduce the psychological, economic, and caregiving burden on families and society, with the goal of enabling some individuals to achieve independent learning, work, and life skills in adulthood.

Research has found that despite social and emotional impairments in daily life, many children with ASD exhibit a great interest in music and some even demonstrate extraordinary musical perception and perfect pitch. When listening to joyful or sad music, children with ASD show activation of cortical and subcortical brain regions, which can enhance their sense of participation in learning and social activities, making music therapy a breakthrough in rehabilitation training and treatment (9, 10).

Geretsegger et al. performed empirical trials to investigate the effectiveness of music therapy in children with ASD, the results confirmed the significant advantages of music therapy as a supplementary modality for treating and educating children with ASD (11). Music therapy facilitates increased communication and social interaction between ASD children and typically developing children, promote social and communication skills development, and facilitate their gradual recovery and successful reintegration into society and family. Although the depth of understanding and frequency of using music therapy in China has been gradually increasing in recent years, how to effectively and feasibly intervene with special children, such as those with ASD, using music therapy remains a challenging problem. Additionally, empirical intervention research on music therapy for ASD children in China is relatively scarce, possibly due to the need for teachers to possess a certain level of professional expertise in music therapy, as well as researchers lacking understanding of how to conduct scientifically valid intervention research.

The Orff music education system is one of the most famous and influential music education systems in the world today, alongside Kodaly’s and Dalcroze’s methods, collectively known as the three most influential music education systems in the world today (12, 13). The teaching strategy of the Orff music education method involves teaching in a small group format in the classroom, combining children’s positive innate performances at different stages of development. It uses singing, speaking, movement, body percussion, and instrument playing to facilitate effective learning processes. Its creative elements include not only sound, but also dance and instrumental music, in order to enhance children’s imagination, strengthen the imitation ability of special children, and create conditions for emotional expansion (12). Ghasemtabar et al. have used Orff music therapy to treat children with ASD and found that it can improve their social interaction and language communication abilities, improve their emotional state, foster interests, and establish good behavioral habits (14). Another study has also found that Orff music therapy can improve auditory processing, language, and cognitive function in children with intellectual disabilities (12). The potential of Orff music therapy to assist in the clinical treatment of children with ASD and enhance treatment efficacy in ASD children has not been adequately investigated in China.

Therefore, this study conducted a clinical trial involving ASD children aged 3–6 years, focusing on practical needs and exploring the clinical effects of Orff music therapy for ASD children from the perspectives of parents, evaluators, and therapists. This study aims to provide theoretical support and clinical evidence for music therapy for ASD children in China.

## 2 Materials and methods

The ethics committees of the Second Affiliated Hospital of Army Medical University reviewed and approved this study, with the Ethics Approval Number: 2024-Researcher No. 143. All subject guardians knew about this study and signed informed consent. All procedures carried out in studies conformed to the 1964 Helsinki Declaration and its subsequent amendments or similar ethical standards.

### 2.1 Research design and study sample

A total of 93 children with ASD who received rehabilitation intervention in the pediatric rehabilitation ward of the author’s hospital from November 2021 to November 2023 were selected. Among them, 45 children received comprehensive rehabilitation training as the control group, and an additional 48 children with ASD received combined interventions of the control group along with Orff music therapy as the observation group. The demographic characteristics of the ASD children were collected, including gender, age, birth weight, gestational age, perinatal asphyxia, maternal prenatal infection, history of epilepsy, parents’ level of education, parents’ age, and monthly family income.

#### 2.1.1 Inclusion criteria

(1) Meet the diagnostic criteria for ASD in the Diagnostic and Statistical Manual of Mental Disorders (DSM-5) (15); (2) Age between 3 to 6 years; (3) Diagnosed by two clinical physicians simultaneously; and (4) Children and their families willing to participate in the questionnaire survey and cooperate with the study procedures.

#### 2.1.2 Exclusion criteria

(1) Children with comorbidities such as hearing impairment, visual impairment, cerebral palsy, and language delay; (2) Intervention received in other medical or educational institutions during the 6-month follow-up period; (3) Parents of the children refuse follow-up visits, assessments, or withdrawal from the intervention before completion; and (4) Children currently participating in other clinical trials.

### 2.2 Assessment

Assessment before and after rehabilitation intervention: the assessment in our study was conducted from three perspectives: parents, therapists, and evaluators. All children with ASD underwent comprehensive assessments using the ABC (parent questionnaire), CRAS (evaluator assessment), and PEP-3 (therapist assessment) prior to intervention, at 3 months post-intervention, and at 6 months post-intervention. In this study, the time of pre-intervention evaluation is defined as T1, the time of 3-month post-intervention review as T2, and the time of 6-month post-intervention review as T3.

#### 2.2.1 Assessment tools

*Autism Behavior Checklist (ABC)* (16): This instrument involves symptoms such as sensation, behavior, emotion, language, and self-care in ASD children. It contains 57 items with a total score of 158. It can be summarized into 5 factors: Sensory (9 items, total score of 26), Relating (12 items, total score of 38), Body and object use (12 items, total score of 38), Language (13 items, total score of 31), and Social and Self help (11 items, total score of 25). Each item is assigned a different score based on its load on the scale ranging from 1 to 4 points. Any child with symptoms will receive a score regardless of severity. The final evaluation results are based on the total scores of all items. The shorter the total score, the better the screening result. If the total score is between 53 and 67, it is considered positive; if it is above 68, it can be used to assist in the diagnosis of autism. The longer the child’s parents or other people who have lived with them for more than 2 weeks participate in the evaluation, which takes about 10–15 min.

*Childhood Autism Rating Scale (CARS)* (17): This instrument includes 15 items related to social relationships, imitation, emotions, physical movements, adaptability, visual responses, auditory responses, olfactory responses, language communication and intelligence. The evaluators score each item based on the strangeness, frequency, severity, and duration of behavior observed through observation and questioning. A four-level scoring method is used with scores ranging from 1 to 4 points depending on age. The maximum score is 60 points. A score below 30 points is considered non-ASD; a score equal to or above 36 points with at least 5 items scoring above 3 points is considered severe ASD; a score between 30 and 36 points with less than 5 items scoring below 3 points is considered mild-to-moderate ASD.

Psycho-educational Profile-3rd edition (PEP-3) was developed by Schopler at North Carolina State University, the Chinese version of PEP-3 was formulated by the Hong Kong Sheng Kung Hui (18). This assessment tool is used to evaluate the developmental level, adaptability, and behavioral characteristics of children with ASD Therapists use the PEP-3 to assess the rehabilitation progress of the patients. The assessment results assist physicians and therapists in determining the appropriate treatment type, intensity, and duration for children with ASD. It provides reference and basis for developing rehabilitation plans to maximize symptom improvement. The assessment consists of two parts: the Developmental and Behavioral sub tests. The Developmental sub tests include Cognitive (Verbal/Preverbal) (CVP), Expressive Language (EL), Receptive Language (RL), Fine Motor (FM), Gross Motor (GM), and Visual-Motor Imitation (VMI). The Behavioral sub tests include Affective Expression (AE), Social Reciprocity (SR), Characteristic Motor Behavior (CMB), and Characteristic Verbal Behavior (CVB). PEP-3 utilizes original scoring to assess the developmental abilities and behavioral characteristics of children with ASD, where higher scores indicate better abilities in children with ASD (19).

### 2.3 Intervention

#### 2.3.1 Control group

Comprehensive rehabilitation intervention was applied, including the following approaches: 1. sensory system training: exercises such as kangaroo jumps and swinging were used to enhance the stability of posture and balance, as well as improve attention and balance abilities; 2. language training: sequential training was provided for deep breathing, language comprehension, expressive language, and language delay; 3. Activities of daily living training: guidance was given to engage the children in daily behavior training, utilizing backward chaining methods. Positive reinforcement, either material or psychological rewards, were given to strengthen desired behaviors if the children showed good completion; 4. social interaction training: games involving physical contact, toy interventions, and role swapping were used to enhance the awareness of social interaction with others; 5. family guidance: parents were advised to provide a relaxed and comfortable home environment, increase physical and verbal engagement with the children, strengthen their attention, improve their behavior, and maintain a balanced diet. Sensory integration training was conducted for 30 min per session, 5 times a week; language training was conducted for 30 min per session, 5 times a week; social interaction training and activities of daily living training were conducted for 30 min per session, 5 times a week; family guidance was provided for 1 h per session, once a week. After 3 months of rehabilitation intervention and based on the results of the PEP-3 assessment, the rehabilitation treatment plan was adjusted, and then the rehabilitation intervention was continued for an additional 3 months.

#### 2.3.2 Observation group

The observation group received the same comprehensive rehabilitation intervention as the control group, along with Orff music therapy. In our medical institution, the introduction of Orff music therapy took place after April 2022. Our research team reviewed the intervention training lesson plans of the patients during their enrollment period. Through checking, it was found that compared with the control group interventions, the Orff music therapists were dedicated professionals who did not undertake other intervention training, thus minimizing the confounding factors between the two groups. The therapy sessions followed a four-stage teaching process of “imitation-imagination-creation-reflection.” Each intervention session started with imitation-based experiential games and ended with activities that encouraged free creativity, such as improvised performances, free discussions, or artistic expressions. Although the program followed specific strategies, the structure and application were carried out within an open and flexible framework, allowing dynamic adjustments based on the ideas and initiatives of the children with ASD. The specific teaching content was divided into three aspects based on the children’s age and their auditory and language developmental characteristics: (1) rhythm training: children were exposed to simple sounds (such as animal sounds and trumpet sounds) for auditory training, and familiar figures and objects (such as names and vehicle) were incorporated into rhythmic nursery rhymes or poems for rhythm training; (2) body movement instruction: teaching was conducted using body percussion, children coordinated their bodies with different tones and rhythms, engaging in activities such as clapping, finger-snapping, thigh-slapping, and foot-stomping, to enhance their coordination of body movements with rhythm; and (3) music instrument instruction: rhythmic, non-pitched percussion instruments, such as drumsticks, wood blocks, chimes, sand hammers, clappers, and triangles, were used to develop children’s sense of rhythm, musicality, and auditory abilities. Orff music therapy was conducted twice a week, with each session lasting 40 min for a group of five children with ASD, the language development quotient, cognitive development quotient, and social development quotient of the groups of 5 children with ASD were matched. After 3 months of rehabilitation intervention, the rehabilitation treatment plan was adjusted based on the results of the PEP-3 assessment, followed by another 3 months of continued rehabilitation intervention. We provided the Supplementary materials of Orff music therapy plan, which includes two examples: Orff Music Therapy Course A and Orff Music Therapy Course B. Course A is the primary version of the treatment plan, while Course B is an upgraded version adjusted by the therapist according to the classroom performance of the children with ASD.

### 2.4 Quality control

All developmental and behavioral pediatricians, children’s healthcare doctors, evaluators, and therapists involved in the study received standardized training. All children with ASD participating in the study were interviewed and diagnosed by experienced developmental and behavioral pediatricians and children’s healthcare doctors using established protocols. Assessments were conducted by qualified assessors who had received training and obtained certification to administer the relevant assessment tools. The assessments took place in the evaluation rooms of the pediatric outpatient department and rehabilitation ward. Comprehensive rehabilitation interventions for both the observation and control groups were carried out by speech therapists, occupational therapists, and physical therapists who had received standardized training. Orff music therapy was conducted by therapists who possessed qualifications as both music teachers and occupational therapists. The rehabilitation interventions were conducted in the therapy rooms of our pediatric rehabilitation ward.

### 2.5 Statistical analysis

Data analysis and graphing were performed using GraphPad Prism 8 statistical software. Descriptive statistics were employed for categorical data and presented as frequencies and percentages, to compare differences between groups, we utilized the χ2 test. Mean ± standard deviation (SD) was used for continuous data, and the *t*-test was employed for group comparisons. *p* < 0.05 was considered statistically significant.

## 3 Results

### 3.1 Population characteristics of included children

**Figure 1:**
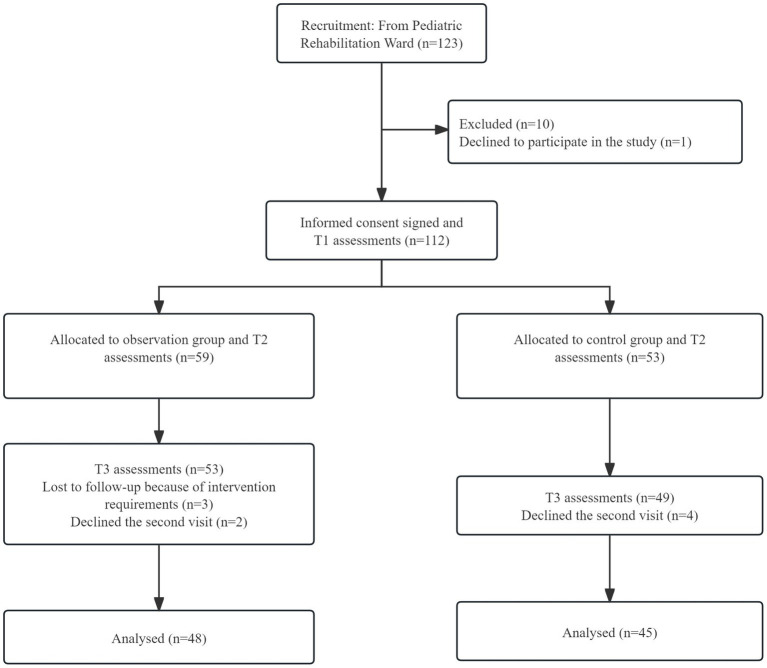
The flowchart of subject enrollment for the two groups of ASD children. T1, pre-intervention; T2, post-intervention at 3 months; T3, post-intervention at 6 months.

A total of 93 children with ASD were included in the study, including 40 in the control group and 43 in the observation group. The flowchart of subject enrollment for the two groups of ASD children is shown in Figure 1. There were no statistically significant differences between the two groups in terms of gender, age, birth weight, gestational age, perinatal asphyxia, prenatal infection status of the mother, history of epilepsy in the child, educational level of the parents, age of the parents, and monthly income of the family (all *p* > 0.05). Table 1 provides the demographic characteristics of the two groups of ASD children. Similarly, there were no statistically significant differences in ABC, CARS, and PEP-3 scores between the two groups of children before the intervention (all *p* > 0.05), as shown in Table 2.

**Table 1:** Baseline demographic characteristics for observation and control groups (*n*, %; x̅ ± s).

Variables	Control group (*n* = 45)	Observation group (*n* = 48)	χ 2/t	*p*
Gender			0.431	0.511
Male	30 (66.7%)	35 (72.9%)		
Female	15 (33.3%)	13 (27.1%)		
Antenatal infection			0.180	0.671
Yes	7 (15.56%)	6 (12.50%)		
No	38 (84.44%)	42 (87.50%)		
Perinatal asphyxia			0.232	0.630
Yes	4 (8.89%)	3 (6.25%)		
No	41 (91.11%)	45 (93.5%)		
Epilepsy			0.415	0.519
Yes	3 (6.67%)	5 (10.42%)		
No	42 (93.33%)	43 (89.58%)		
Education of mother			0.784	0.676
Junior high school	8 (17.78%)	6 (12.50%)		
Senior high school	7 (15.56%)	10 (20.83%)		
≥University	30 (66.67%)	32 (66.67%)		
Education of father			0.173	0.917
Junior high school	10 (22.22%)	9 (18.75%)		
Senior high school	10 (22.22%)	11 (22.92%)		
≥University	25 (55.56%)	28 (58.33%)		
Family’s monthly income (RMB)			0.522	0.770
≤5,000	10 (22.22%)	8 (16.67%)		
5,000< ~ ≤1,000	20 (4.44%)	24 (50.00%)		
≥10,000	15 (33.33%)	16 (33.33%)		
Baseline age (months)	54.22 + 10.61	54.82 + 11.19	0.265	0.792
Paternal age (years)	33.26 + 6.46	33.61 + 5.52	0.282	0.779
Maternal age (years)	29.39 + 5.17	30.16 + 5.14	0.720	0.473
Gestational age (weeks)	38.90 + 1.87	38.77 + 2.08	0.316	0.753
Birth weight (kg)	3.57 + 0.50	3.55 + 0.57	0.179	0.858

**Table 2:** The ABC, CARS, and PEP-3 scores in ASD children before and after intervention (x̅ ± s).

	T1		Control group (*n* = 45)			Observation group (*n* = 48)		
	*t*	*p*	T1	T2	T3	T1	T2	T3
ABC								
S	0.259	0.796	20.60 + 2.41	18.64 + 2.26	16.80 + 1.71	20.73 + 2.43	16.71 + 2.37	13.60 + 1.78
R	0.620	0.537	26.64 + 3.18	25.02 + 2.60	23.40 + 2.82	27.02 + 2.73	23.31 + 2.74	20.65 + 2.24
B	0.449	0.654	14.87 + 3.87	13.78 + 3.12	12.69 + 2.58	14.54 + 4.20	13.06 + 3.44	10.93 + 2.35
L	0.545	0.587	25.18 + 2.43	23.11 + 2.40	20.51 + 2.21	25.44 + 2.17	21.71 + 2.55	17.90 + 2.56
S-S	0.874	0.384	14.84 + 3.16	13.87 + 2.44	11.62 + 1.81	15.44 + 3.44	14.17 + 2.79	11.23 + 1.81
T	0.343	0.732	102.13 + 14.78	94.42 + 11.83	85.02 + 8.42	103.17 + 14.42	88.96 + 11.40	74.31 + 7.39
PEP-3								
CVP	0.396	0.693	21.36 + 7.92	26.80 + 9.32	31.42 + 9.78	21.98 + 7.16	26.73 + 9.15	36.23 + 8.51
EL	0.372	0.711	20.38 + 8.98	25.69 + 10.12	30.47 + 9.78	19.69 + 8.89	29.04 + 10.80	37.50 + 8.80
RL	0.327	0.745	16.40 + 6.46	20.82 + 6.28	23.73 + 6.90	15.98 + 5.93	22.98 + 6.59	28.13 + 5.85
FM	1.070	0.287	23.76 + 5.42	25.93 + 6.14	29.40 + 5.35	22.54 + 5.56	25.81 + 6.02	31.98 + 5.34
GM	0.141	0.888	22.22 + 3.43	23.51 + 3.30	24.67 + 3.23	22.33 + 4.05	24.02 + 4.82	25.65 + 4.36
VMI	1.211	0.229	9.36 + 2.28	11.11 + 2.77	12.42 + 3.07	8.75 + 2.56	11.19 + 2.94	13.58 + 2.80
AE	1.054	0.295	13.76 + 1.72	14.91 + 1.83	15.80 + 1.71	13.35 + 2.01	15.06 + 1.92	17.06 + 1.71
SR	0.130	0.897	8.84 + 2.12	10.09 + 2.73	11.49 + 2.86	8.90 + 2.31	11.33 + 2.89	13.96 + 3.08
CMB	0.968	0.336	11.71 + 2.50	12.44 + 2.71	13.09 + 2.69	12.21 + 2.48	13.04 + 2.78	14.06 + 2.52
CVB	0.535	0.594	8.64 + 2.53	9.58 + 3.22	10.27 + 3.03	8.96 + 3.18	10.29 + 3.44	11.31 + 3.11
CARS	1.645	0.104	36.57 + 2.91	34.20 + 2.27	33.00 + 1.67	37.46 + 2.29	34.15 + 1.97	32.29 + 1.41

ABC, autism behavior checklist; CARS, childhood autism rating scale; PEP-3, psycho-educational profile-3rd edition; ASD, autism spectrum disorder; S, sensory; R, relating; B, body and object use; L, language; S-S, social and self help; T, total score of ABC; CVP, cognitive (verbal/preverbal); EL, expressive language; RL, receptive language; FM, fine motor; GM, gross motor; VMI, visual motor imitation; AE, affective expression; SR, social reciprocity; CMB, characteristic motor behavior; CVB, characteristic verbal behavior; T1, pre-intervention; T2, post-intervention at 3 months; T3, post-intervention at 6 months.

### 3.2 Comparison of ABC scores in ASD children before and after intervention

**Figure 2:**
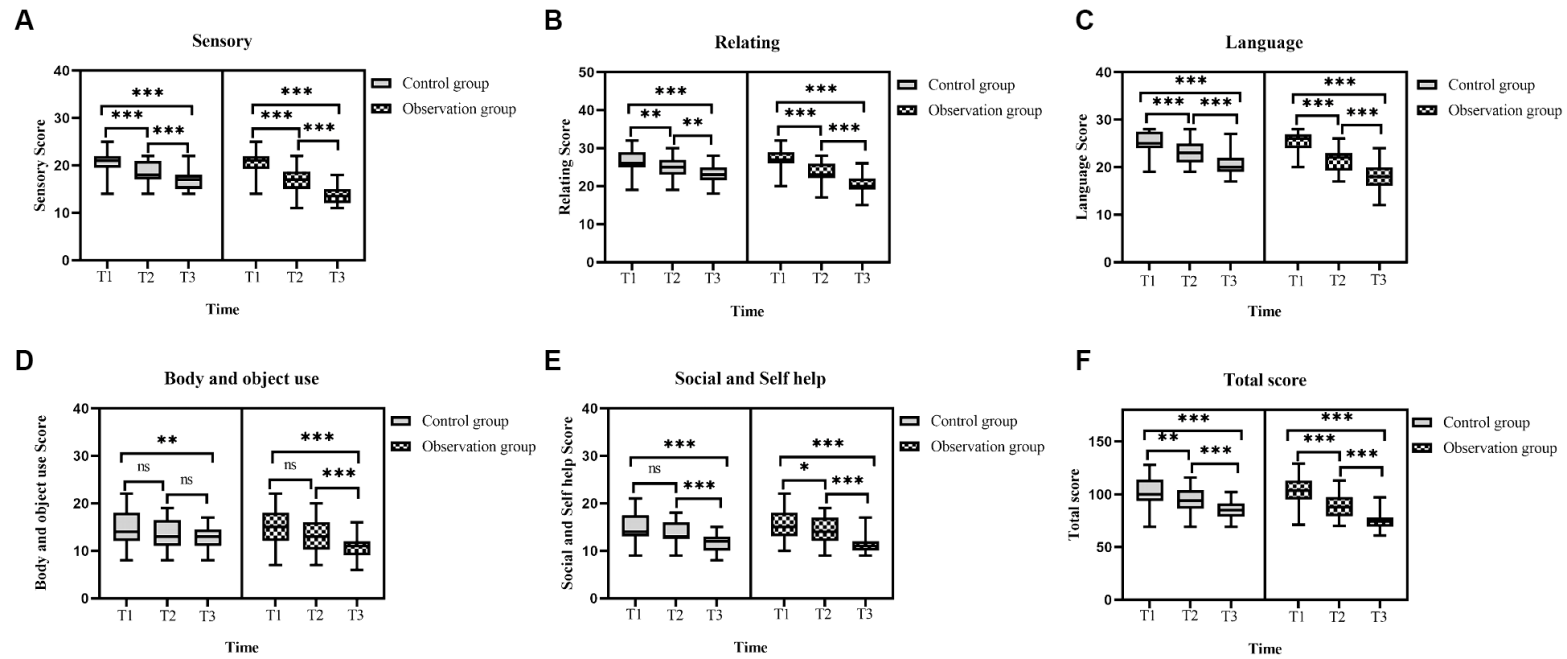
Comparison of the ABC scores in ASD children before and after intervention. **(A)** The sensory scores in the two groups of ASD children before and after intervention. **(B)** The relating scores in the two groups of ASD children before and after intervention. **(C)** The language scores in the two groups of ASD children before and after intervention. **(D)** The body and object use scores in the two groups of ASD children before and after intervention. **(E)** The social and self help scores in the two groups of ASD children before and after intervention. **(F)** The total score of ABC in the two groups of ASD children before and after intervention. ABC, autism behavior checklist; ASD, autism spectrum disorder; T1, pre-intervention; T2, post-intervention at 3 months; T3, post-intervention at 6 months; ns: >0.05; *: <0.05; **: <0.01; ***: <0.001.

Compared at the T1, T2, and T3 time points, there was a gradual decrease trend in the Sensory, Relating, Language, and ABC total scores of the two groups of ASD children (Figures 2A–C,F), and the differences were statistically significant, both within the observation group and the control group of ASD children (T1 vs. T2, T2 vs. T3, T1 vs. T3, all *p* < 0.001). Although in terms of Body and object use and Social and self help, the scores of both groups of ASD children also showed a gradual decrease trend (Figures 2D,E), the statistical significance of the scores at different time points was different. For the observation group of ASD children (T1 vs. T2, T2 vs. T3, T1 vs. T3), the Body and object use (*p* = 0.062 and *p* < 0.001 and *p* < 0.001) and the Social and self help (*p* = 0.05 and *p* < 0.001 and *p* < 0.001); while for the control group of ASD children (T1 vs. T2, T2 vs. T3, T1 vs. T3), Body and object use (*p* = 0.145 and *p* = 0.074 and *p* = 0.002) and Social and self help (*p* = 0.107 and *p* < 0.001 and *p* < 0.001). Detailed data of the ABC for the two groups are shown in Table 2. From the parents’ perspective, short-term intervention of 3 months had clinical effects on Sensory, Relating and Language for both groups of ASD children. Body and object use improved significantly after 3 months of intervention for the observation group of ASD children, while it took 6 months of continuous intervention for the control group of ASD children to show significant clinical effects. In terms of Social and self help, both groups of ASD children required continuous intervention for 6 months to see improvement.

### 3.3 Comparison of CARS scores in ASD children before and after intervention

**Figure 3:**
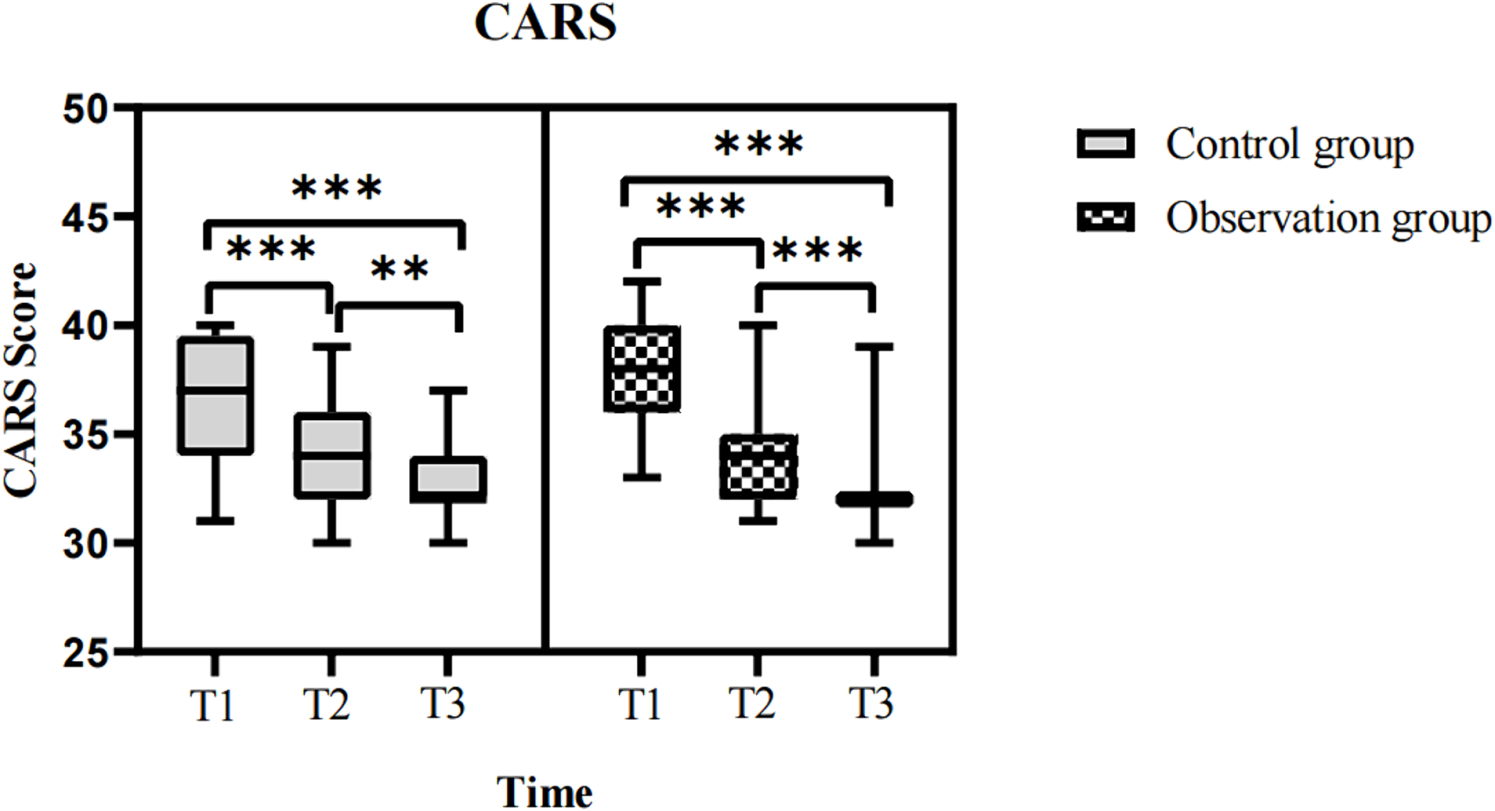
Comparison of the CARS scores in ASD children before and after intervention. CARS, childhood autism rating scale; ASD, autism spectrum disorder; T1, pre-intervention; T2, post-intervention at 3 months; T3, post-intervention at 6 months; ns: >0.05; *: <0.05; **: <0.01; ***: <0.001.

When comparing at T1, T2, and T3 time points, both groups of children with ASD showed a gradual decrease trend in CARS scores (Figure 3), and the differences were statistically significant. For ASD children, the CARS scores (T1 vs. T2, T2 vs. T3, T1 vs. T3) in the observation group were (*t* = 7.591, *p* < 0.001 and *t* = 5.319, *p* < 0.001 and *t* = 13.319, *p* < 0.001), while in the control group, the scores were (*t* = 5.491, *p* < 0.001 and *t* = 2.857, *p* < 0.001 and *t* = 8.834, *p* < 0.001). Detailed data on the CARS scores of both groups are presented in Table 2. From the perspective of the evaluators, there was a significant decrease in CARS scores for both groups of ASD children after 3 months of intervention, and the effects persisted until 6 months of intervention. This indicates that both individual comprehensive rehabilitation intervention and combined Orff music therapy have clinical effects on the treatment of ASD children in the short-term intervention of 3 months.

### 3.4 Comparison of PEP-3 scores in ASD children before and after intervention

**Figure 4:**
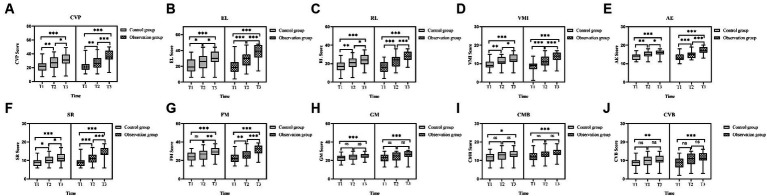
Comparison of the PEP-3 scores in ASD children before and after intervention. **(A)** The CVP scores in the two groups of ASD children before and after intervention. **(B)** The EL scores in the two groups of ASD children before and after intervention. **(C)** The RL scores in the two groups of ASD children before and after intervention. **(D)** The VMI scores in the two groups of ASD children before and after intervention. **(E)** The AE scores in the two groups of ASD children before and after intervention. **(F)** The SR scores in the two groups of ASD children before and after intervention. **(G)** The FM scores in the two groups of ASD children before and after intervention. **(H)** The GM scores in the two groups of ASD children before and after intervention. **(I)** The CMB scores in the two groups of ASD children before and after intervention. **(J)** The CVB scores in the two groups of ASD children before and after intervention. PEP-3, psycho-educational profile-3rd edition; ASD, autism spectrum disorder; CVP, cognitive (verbal/preverbal); EL, expressive language; RL, receptive language; FM, fine motor; GM, gross motor; VMI, visual motor imitation; AE, affective expression; SR, social reciprocity; CMB, characteristic motor behavior; CVB, characteristic verbal behavior; T1, pre-intervention; T2, post-intervention at 3 months; T3, post-intervention at 6 months; ns: >0.05; *: <0.05; **: <0.01; ***: <0.001.

At T1, T2, and T3 time points, the CVP, EL, RL, VMI, AE, and SR scores in the PEP-3 assessments showed a progressively increasing trend in two groups of the children with ASD (Figures 4A–F), and the differences were statistically significant (T1 vs. T2, T2 vs. T3, T1 vs. T3, *p* < 0.05). Although FM, GM, CMB, and CVB scores also showed a progressively increasing trend in both groups (Figures 4G–J), the statistical significance of the scores at different time points was different. For the observation group (T1 vs. T2, T2 vs. T3, T1 vs. T3), the FM scores were (*p* = 0.007 and *p* < 0.001 and *p* < 0.001), GM scores were (*p* = 0.066 and *p* = 0.086 and *p* < 0.001), CMB scores were (*p* = 0.126 and *p* = 0.063 and *p* < 0.001), and CVB scores were (*p* = 0.052 and *p* = 0.131 and *p* < 0.001); While in the control group (T1 vs. T2, T2 vs. T3, T1 vs. T3), the FM scores were (*p* = 0.079 and *p* = 0.005 and *p* < 0.001), GM scores were (*p* = 0.073 and *p* = 0.096 and *p* < 0.001), CMB scores were (*p* = 0.188 and *p* = 0.257 and *p* = 0.014), and CVB scores were (*p* = 0.127 and *p* = 0.298 and *p* = 0.007). The detailed data of PEP-3 scores in both groups are shown in Table 2. From the perspective of therapists, CVP, EL, RL, VMI, AE, and SR of both groups of ASD children showed clinical effects after a short-term intervention of 3 months, and these effects were sustained for 6 months. In terms of FM, the observation group ASD children showed significant improvement after a 3-month intervention, and this improvement continued for 6 months, while the control group ASD children required a continuous intervention for 6 months to achieve noticeable clinical effects. In terms of GM, CMB (e.g., transitioning actions, using visual cues), and CVB (e.g., active communication, sustained conversation abilities), both groups of ASD children required a continuous intervention of 6 months to achieve significant clinical effects.

### 3.5 Comparison of the changes in ABC scores at different time intervals (T1–T2, T2–T3, and T1–T3) between two groups of children with ASD

**Figure 5:**
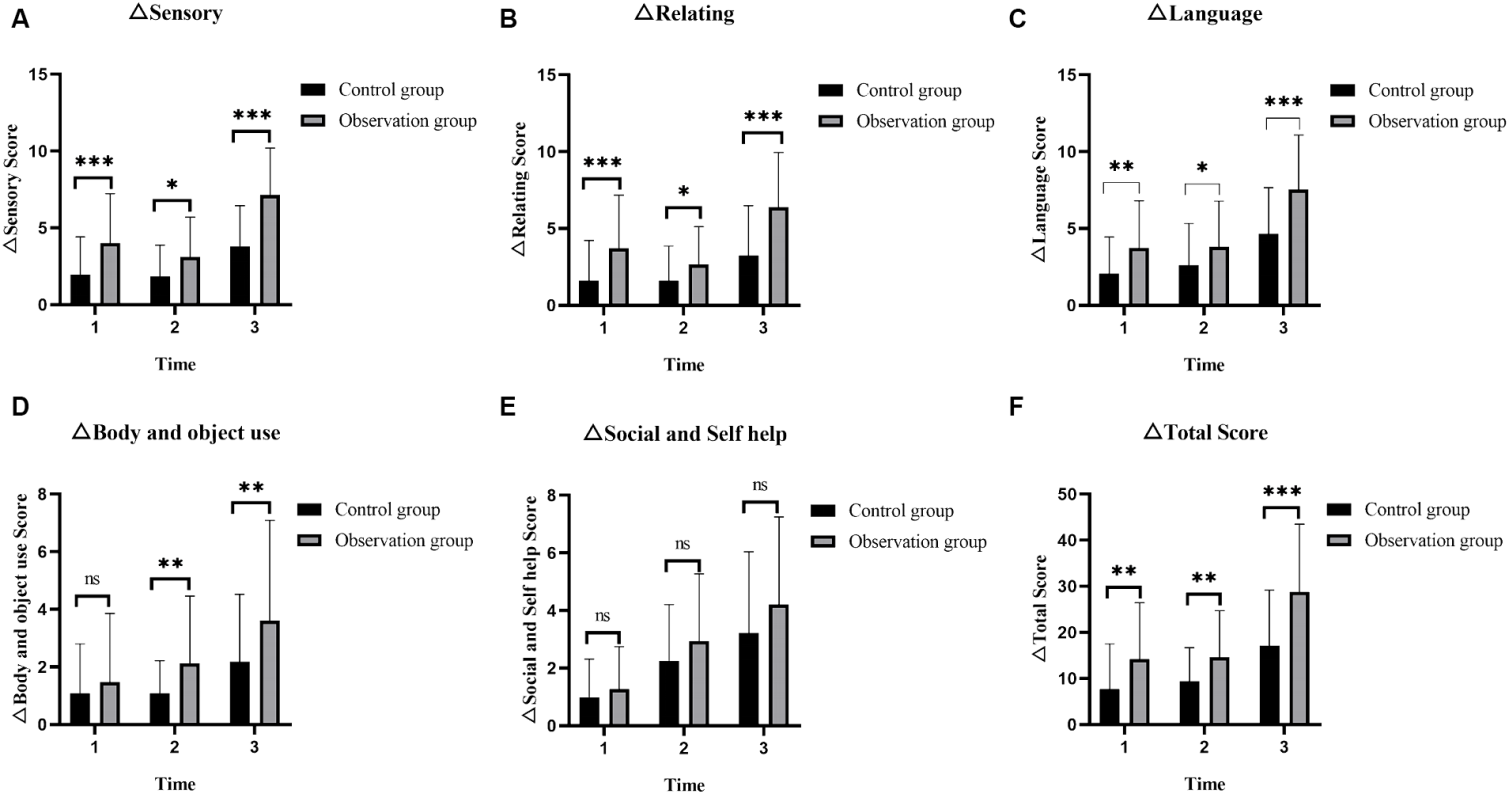
The changes in ABC scores at different time intervals (T1–T2, T2–T3, and T1–T3) between two groups of the children with ASD. **(A)** The changes of sensory scores in the two groups at different time intervals. **(B)** The changes of relating scores in the two groups at different time intervals. **(C)** The changes of language scores in the two groups at different time intervals. **(D)** The changes of body and object use scores in the two groups at different time intervals. **(E)** The changes of social and self help scores in the two groups at different time intervals. **(F)** The changes of total scores in the two groups of ASD children before and after intervention. ABC, autism behavior checklist; ASD, autism spectrum disorder; 1: T1–T2 = The time interval from pre-intervention to post-intervention at 3 months; 2: T2–T3 = The time interval from post-intervention at 3 months to post-intervention at 6 months; 3: T1–T3 = The time interval from pre-intervention to post-intervention at 6 months; ns: >0.05; *: <0.05; **: <0.01; ***: <0.001.

Research showed that the scores for Sensory, Relating, Language, and ABC total score in the ASD children of the observation group were significantly lower than those of the control group in the three time periods of T1–T2, T2–T3, and T1–T3 (all *p* < 0.05) (Figures 5A–C,F). When comparing with the control group, the score reduction in Body and object use of the ASD children in the observation group showed no significant advantage in the T1–T2 time period (*p* = 0.429), while in the T2–T3 and T1–T3 time periods, the score reduction was significantly better than that of the control group (*p* = 0.008 and *p* = 0.019) (Figure 5D). The reduction in Social and self help, whether in the T1–T2 or T2–T3 time period, or even in the T1–T3 time period, did not differ significantly between the two groups (*p* = 0.324, *p* = 0.128, and *p* = 0.107) (Figure 5E). The detailed data of the reduction in ABC scores for each time interval are presented in Table 3. Therefore, based on the parental perspective, Orff music therapy has shown a significant positive effect on sensory, relating and language in ASD children, while the positive effect on Body and object use requires a certain amount of accumulated time for Orff music therapy to manifest, and Orff music therapy does not significantly improve the Social and self help of ASD children.

**Table 3:** The changes in ABC, CARS, and PEP-3 scores at different time intervals between two groups of the children with ASD (x̅ ± s).

	Control group (*n* = 45)			Observation group (*n* = 48)		
	T1–T2	T2–T3	T1–T3	T1–T2	T2–T3	T1–T3
ABC						
S	1.96 + 2.47	1.84 + 2.03	3.80 + 2.64	4.02 + 3.20	3.10 + 2.59	7.13 + 3.08
R	1.62 + 2.60	1.62 + 2.24	3.24 + 3.24	3.71 + 2.46	2.67 + 2.45	6.38 + 3.56
B	1.48 + 2.38	2.13 + 2.34	3.60 + 3.49	1.09 + 2.35	1.09 + 1.12	2.12 + 2.35
L	2.07 + 2.38	2.60 + 2.73	4.67 + 2.99	3.73 + 3.08	3.81 + 2.97	7.54 + 3.54
S-S	0.98 + 1.34	2.24 + 1.96	3.22 + 2.81	1.27 + 1.47	2.93 + 2.34	4.21 + 3.04
T	7.71 + 9.82	9.40 + 7.30	17.11 + 12.02	14.21 + 12.18	14.65 + 10.03	28.85 + 14.61
PEP-3						
CVP	5.44 + 7.64	4.62 + 7.75	10.07 + 9.74	4.75 + 6.65	9.50 + 9.44	14.25 + 9.57
EL	5.31 + 7.54	4.78 + 7.84	10.09 + 9.48	9.35 + 9.80	8.46 + 8.74	17.81 + 10.05
RL	4.42 + 4.96	2.91 + 4.36	7.33 + 6.21	7.00 + 6.76	5.15 + 5.91	12.15 + 7.52
FM	2.18 + 3.40	3.47 + 6.09	5.64 + 6.58	3.27 + 3.81	6.17 + 6.23	9.44 + 6.52
GM	1.29 + 1.65	1.16 + 1.11	2.44 + 1.87	1.69 + 2.06	1.63 + 3.14	3.31 + 3.20
VMI	1.76 + 2.44	1.31 + 2.07	3.07 + 2.02	2.44 + 2.95	2.40 + 2.89	4.83 + 3.41
AE	1.16 + 1.55	0.89 + 1.58	2.04 + 1.89	1.71 + 1.86	2.00 + 2.07	3.71 + 2.31
SR	1.24 + 2.05	1.40 + 2.32	2.64 + 3.06	2.44 + 2.34	2.63 + 3.13	5.04 + 3.22
CMB	0.73 + 1.48	0.64 + 1.73	1.38 + 2.08	0.83 + 1.48	1.02 + 2.29	1.85 + 2.44
CVB	0.93 + 1.42	0.69 + 2.14	1.62 + 2.34	1.33 + 1.97	1.02 + 2.25	2.31 + 2.88
CARS	2.38 + 2.24	1.20 + 1.29	3.58 + 2.33	3.29 + 1.84	1.85 + 1.47	5.17 + 1.99

ABC, autism behavior checklist; CARS, childhood autism rating scale; PEP-3, psycho-educational profile-3rd edition; S, sensory; R, relating; B, body and object use; L, language; S-S, social and self help; T, total score of ABC; CVP, cognitive (verbal/preverbal); EL, expressive language; RL, receptive language; FM, fine motor; GM, gross motor; VMI, visual motor imitation; AE, affective expression; SR, social reciprocity; CMB, characteristic motor behavior; CVB, characteristic verbal behavior; T1–T2, The time interval from pre-intervention to post-intervention at 3 months; T2–T3, The time interval from post-intervention at 3 months to post-intervention at 6 months; T1–T3, The time interval from pre-intervention to post-intervention at 6 months.

### 3.6 Comparison of the changes in CARS scores at different time intervals (T1–T2, T2–T3, and T1–T3) between two groups of children with ASD

**Figure 6:**
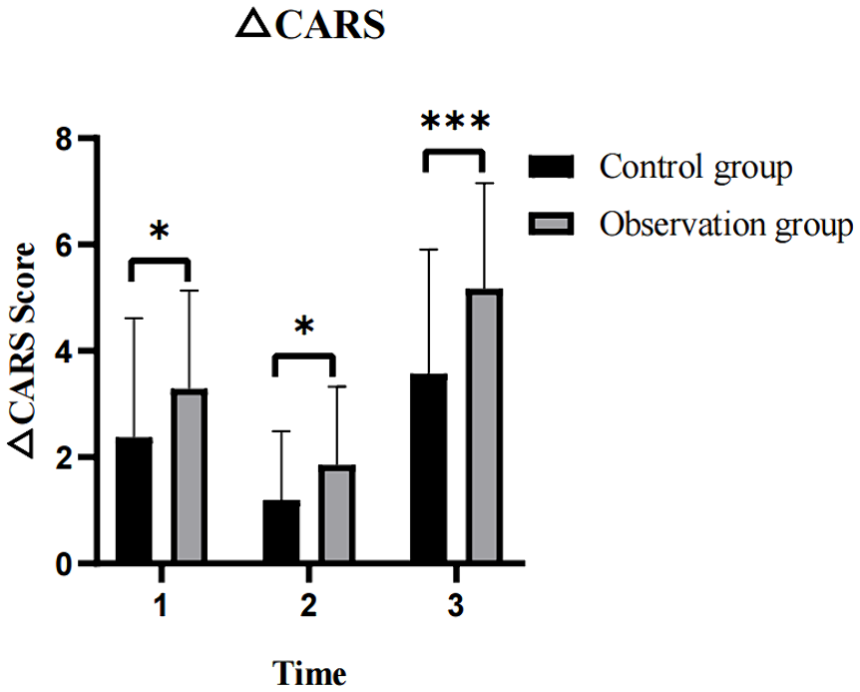
The changes in CARS scores at different time intervals (T1–T2, T2–T3, and T1–T3) between two groups of the children with ASD. CARS, childhood autism rating scale; ASD, autism spectrum disorder; 1: T1–T2 = The time interval from pre-intervention to post-intervention at 3 months; 2: T2–T3 = The time interval from post-intervention at 3 months to post-intervention at 6 months; 3: T1–T3 = The time interval from pre-intervention to post-intervention at 6 months; ns: >0.05; *: <0.05; **: <0.01; ***: <0.001.

The observation group ASD children showed a significantly higher reduction in CARS scores during the three time intervals T1–T2, T2–T3, and T1–T3 compared to the control group, and the differences were statistically significant (*t* = 2.146, *p* = 0.035 and *t* = 2.260, *p* = 0.026 and *t* = 46, *p* < 0.001) (Figure 6). The detailed data can be found in Table 3. From the perspective of evaluators, Orff music therapy can alleviate the condition of children with ASD and reduce the severity of the disease.

### 3.7 Comparison of the changes in PEP-3 scores at different time intervals (T1–T2, T2–T3, and T1–T3) between two groups of children with ASD

**Figure 7:**
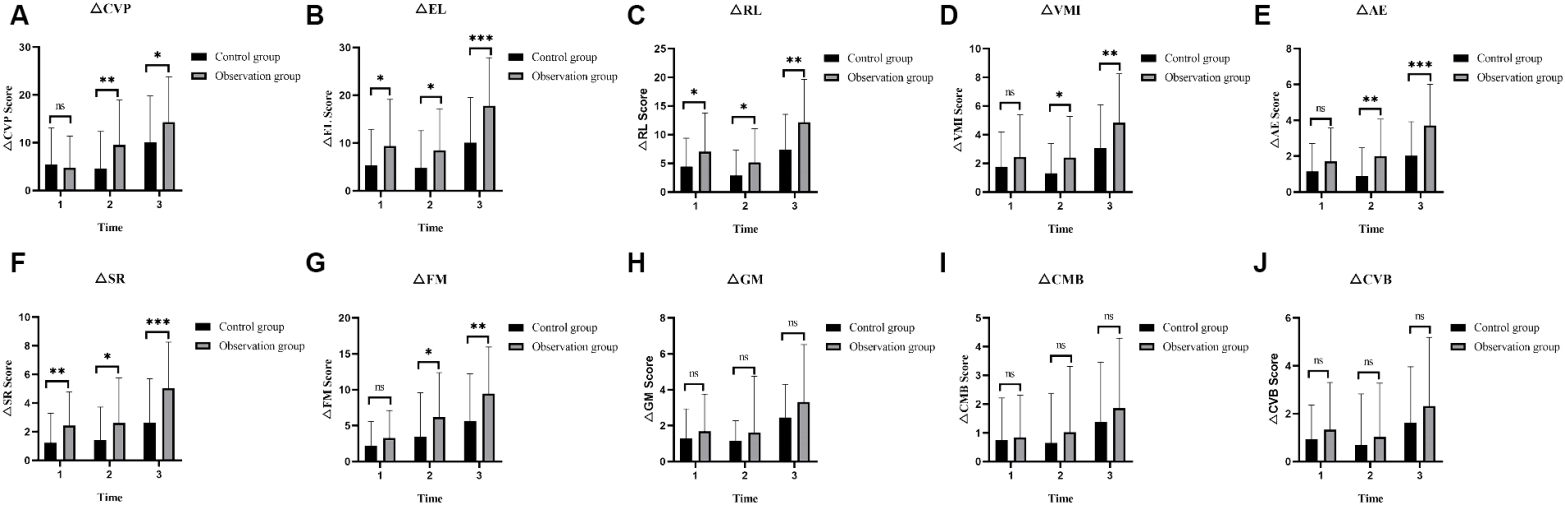
The changes in PEP-3 scores at different time intervals (T1–T2, T2–T3, and T1–T3) between two groups of the children with ASD. **(A)** The changes of CVP scores in the two groups at different time intervals. **(B)** The changes of EL scores in the two groups at different time intervals. **(C)** The changes of RL scores in the two groups at different time intervals. **(D)** The changes of VMI scores in the two groups at different time intervals. **(E)** The changes of AE scores in the two groups at different time intervals. **(F)** The changes of SR scores in the two groups at different time intervals. **(G)** The changes of FM scores in the two groups at different time intervals. **(H)** The changes of GM scores in the two groups at different time intervals. **(I)** The changes of CMB scores in the two groups at different time intervals. **(J)** The changes of CVB scores in the two groups at different time intervals. PEP-3, psycho-educational Profile-3rd edition; ASD, autism spectrum disorder; CVP, cognitive (verbal/preverbal); EL, expressive language; RL, receptive language; FM, fine motor; GM, gross motor; VMI, visual motor imitation; AE, affective expression; SR, social reciprocity; CMB, characteristic motor behavior; CVB, characteristic verbal behavior; 1: T1–T2 = The time interval from pre-intervention to post-intervention at 3 months; 2: T2–T3 = The time interval from post-intervention at 3 months to post-intervention at 6 months; 3: T1–T3 = The time interval from pre-intervention to post-intervention at 6 months; ns: >0.05; *: <0.05; **: <0.01; ***: <0.001.

In the observation group, the score improvements in EL, RL, and SR of PEP-3 assessment for ASD children were significantly higher than those of the control group in the three time intervals T1–T2, T2–T3, and T1–T3, with statistically significant differences (all *p* < 0.05) (Figures 7B,C,F). Although there were no significant differences in the score improvement levels of CVP, FM, VMI, and AE between the two groups in the time interval T1–T2 (all *p* > 0.05), the score improvement levels were significantly higher than the control group in the time intervals T2–T3 and T1–T3 (all *p* < 0.05) (Figures 7A,D,E,G). There were no statistically significant differences in the score improvement levels of GM, CMB, and CVB between the two groups of ASD children in the three time intervals T1–T2, T2–T3, and T1–T3 (all *p* > 0.05) (Figures 7H–J). Detailed data on the scores of each factor of PEP-3 in the two groups of ASD children are shown in Table 3. From the perspective of therapists, Orff music therapy can significantly improve the language expression, language comprehension, and social interaction of ASD children in rehabilitation intervention therapy after 3 months. Only after 6 months of continuous intervention, it can significantly improve the cognitive ability, fine motor skills, imitation ability, and emotional expression of ASD children, while it does not have a significant improvement effect on the characteristic motor behavior of ASD children (e.g., the use of taste and visual abilities, reactions to sound) and the characteristic verbal behavior (e.g., adjusting tone, volume, and speed during speech).

## 4 Discussion

ASD is a common and severe developmental disorder characterized by core symptoms such as social communication difficulties, abnormal behavior and emotions, restricted interests, and impaired emotion recognition. Without timely intervention, ASD can not only affect the developmental progress of affected children but also significantly impact their quality of life. Orff music therapy is an active participatory teaching method that is based on rhythm training and combines teaching methods of listening, singing, movement, and playing instruments (20). In this teaching approach, children with ASD improve their visual and tactile perception through body movements and music instrument playing to compensate for auditory deficiencies. Rhythm training can enhance the language expression ability of children with ASD, and interaction with peers and instrument playing can improve their social communication skills. Our study shows significant improvements in language comprehension, language expression, and social interaction in children with ASD after 3 months of combined Orff music therapy compared to the control group, and these abilities continue to improve during the T2–T3 time period. Although cognitive abilities, imitation abilities, emotional expression, and fine motor skills did not show significant changes compared to the control group after 3 months of intervention, these abilities were significantly better than the control group after 6 months of intervention. we also have reviewed nearly a decade’s worth of typical early studies, and currently there are few no clinical research reports on Orff music. However, we have retrieved several clinical studies on other types music therapy for children with autism spectrum disorder (ASD). Most of these studies demonstrate that music therapy can significantly improve various clinical manifestations in children with ASD. The relevant information from these studies has been summarized and presented in a table (as shown in Supplementary Table S1).

In our study, both the parent questionnaire ABC scale and the therapist’s PEP-3 evaluation showed that the language expression and language comprehension of children in the observation group were significantly better than those in the control group after 3 months of intervention, and this therapeutic effect continued to improve during the T3–T6 time period. Language communication difficulties are the greatest obstacles for children with ASD, and a lack of motivation for communication is a major factor hindering their language development and behavioral expression ability. Previous studies have reported that music therapy can improve the language expression ability of children with ASD (21). The reason is that music stimuli can activate the auditory areas of the brain, leading to synaptic learning and plasticity changes in this region. At the same time, the music teaching environment can enhance the visual and tactile perception of children with ASD, allowing them to experience music with multiple senses. Listening to music can activate the auditory areas of the brain to improve auditory abilities (21). Research has also shown that the Broca area is a brain region that overlaps music and language processing. The larger the activated brain region, the greater the synaptic learning and plasticity changes in this area. Based on the principles of neural plasticity, language abilities can be improved by activating the brain’s language processing area. Music and language share the same neural processing areas, and stimulation from music can awaken neural development and promote language development (22). Consistent with previous studies, in our study, most children with ASD had limited language skills and did not communicate with others. Our therapists incorporated commonly used language and animal sounds into songs, guided the children through singing and games, and used rhythms and repeated singing to improve pronunciation and tone recognition, expand their vocabulary, and improve their language expression and communication abilities. In addition, in the Orff music therapy, music stories and the use of musical instruments were integrated. For example, in the singing segment, the nursery rhyme “Wow Wow Meow Meow” was introduced for children to recognize pictures of cats and dogs and listen to their corresponding sounds, imitating animal sounds like “woof woof” and “meow meow.” In the dancing segment, the song “The Harvest Song” was used to teach children to imitate morning routines such as waking up, getting dressed, brushing teeth, and washing their faces. Concrete and concise story elements were presented through music, allowing children to listen to, understand, participate in, experience, and enjoy music and stories, thus promoting their language comprehension abilities. Kim et al. reported that during Orff music game teaching, the involvement of playing instruments and creating melodies requires cooperation and turn-taking between therapists and children with ASD, resulting in significantly improved responsivity and joint attention of the participants, further promoting their language development. Non-verbal children with ASD, after the intervention, began to produce simple, monosyllabic vocalizations and showed increased enjoyment and more appropriate response behaviors (23). This is also one of the advantages of using music instruments in Orff music therapy for children with ASD in this study.

The social skills of children with ASD in the observation group significantly improved compared to the control group after 3 months of intervention, and this effect continued until 6 months of intervention. Although the improvement in cognitive abilities, imitation, and emotional expression in the observation group of children with ASD was comparable to the control group during the first 3 months, these abilities were better than the control group after 6 months of intervention. During Orff music therapy in our study, many greeting songs and instruments that promote social interaction were used. For example, through singing songs like “Hello,” “Making a Call,” and “Crossing the Road,” children with ASD learned how to communicate with others through rhymes. This approach involved the participation of parents, therapists, and children, and guided children with ASD to gradually develop confidence and interest in communication, transitioning from being unwilling to communicate with others to being willing to communicate. They learned to shake hands with others, make eye contact, and accept physical contact with others, showing improved social interaction and emotional expression abilities. A study reported that about 95% of the studies included in the review indicated that Orff music teaching significantly improved the social interaction and cognitive abilities of children with ASD (24), and another research has also found that Orff teaching can achieve high social validity, and parents of participants expressed satisfaction with the application of Orff teaching, recognizing its important role in the emotional expression and cognitive abilities of their children. Parents observed improvement in their children’s ability to learn similar concepts and the speed of learning, as well as improved understanding of other colors and communication skills after participating in the study (25). Redondo et al. Reported that child-centered teaching and creative music-making Orff music teaching effectively improved the social interaction and language communication abilities of children with autism spectrum disorder and helped reduce repetitive behaviors (26). Additionally, Kaplan et al. found that Orff music teaching can increase communication between patients and therapists and help children with ASD improve their social skills, imitation abilities, and cognitive abilities (27). Similarly, in this study, a combination of music and movement was used in Orff music education, incorporating movements to promote the overall coordination abilities of ASD children’s bodies, which greatly aids the development of their imitation abilities.

Based on the PEP-3 assessment and therapist observation, the fine motor abilities of children with ASD in our study were significantly superior to those in the control group. Our therapists employed percussion instruments in Orff music therapy for ASD children, taking the teaching of “Crossing the Road” as an example, using tambourine as the music instrument. Through playing the tambourine, students experienced the initiation and cessation of sound and action, sensed the beginning of sound, and felt their bodily control. These actions enhance fine motor abilities, including precise control of the hands, fingers, and arms, known as fine motor skills. Additionally, music instruments enable ASD children to become active participants in the teaching process. They can express themselves through playing music instruments, maintaining a strong interest in music instruments such as the tambourine, producing rhythmic and clear sounds that promote language and cognitive development. Studies have reported a correlation between the development of fine motor skills in ASD children and the development of cognitive and language abilities. Improved fine motor skills facilitate better daily functioning and social interaction for ASD children, as these practical experiences may enhance attention, memory, and other cognitive abilities, thereby promoting cognitive development (28). Furthermore, the improvement of fine motor skills may contribute to better language comprehension and usage, thus promoting language development in ASD children (29, 30). This may be one of the reasons why the introduction of Orff music in this study resulted in improved language abilities in ASD children after 3 months of intervention. Pereira et al. reported that Orff music therapy, which includes circle dances and various body movements, such as rhythmic body percussion (hitting different body parts), walking, running, and jumping in response to music, can promote gross motor development in ASD children (31, 32). In our study, however, the level of improvement in gross motor abilities in the Orff music group did not show significant differences compared to the control group. One possible reason for this discrepancy may be the emphasis on the use of instruments in this study, as the instruments used mainly focused on hand usage rather than gross motor movement. In future clinical treatments, it may be beneficial to incorporate gross motor training into Orff music therapy to enhance balance, limb coordination, strengthen the child’s vestibular proprioception, and expand visual spatial awareness.

## 5 Limitations

The current findings should be considered in the context of several limitations. First, the sample size of this study was relatively small, which may limit the generalizability of the results. Therefore, it is recommended to increase the sample size in future research to obtain more representative results. Additionally, multicenter studies could be considered to further validate the effects of music therapy on children with ASD. Second, this study only used Orff music therapy, and other types of music therapy may have different effects. Therefore, it is suggested to compare the effects of different types of music therapy in future research to determine the most suitable music therapy approach for children with ASD. Third, this study only assessed short-term effects (3 months and 6 months), and it is unclear whether the effects of music therapy will change or persist over a longer period. Therefore, it is recommended to conduct longer follow-up in future research to evaluate the long-term effects of music therapy, providing a more comprehensive understanding of the sustained impact and further development of music therapy in children with ASD.

## 6 Conclusion

In conclusion, this study found that Orff music therapy has positive effects on children with ASD. In the short-term intervention (3 months), there were significant improvements in language expression, language comprehension, and social skills in children with ASD. With 6 months of intervention, there were also significant improvements in cognitive abilities, imitation abilities, emotional expression, and fine motor skills. However, it is important to note that although most symptoms of children with ASD were improved, there were no significant improvements in gross motor skills, characteristic motor behavior and characteristic verbal behavior. This suggests that Orff music therapy may not have a significant impact on these specific developmental domains in children with ASD. Therefore, further research is needed to investigate the long-term effects of music therapy and to compare the effects of different types of music therapy approaches to optimize the strategies for music therapy in children with ASD.

## Data availability statement

The original contributions presented in the study are included in the article/Supplementary material, further inquiries can be directed to the corresponding authors.

## Ethics statement

The ethics committees of the Second Affiliated Hospital of Army Medical University reviewed and approved this study. The studies were conducted in accordance with the local legislation and institutional requirements. Written informed consent for participation in this study was provided by the participants’ legal guardians/next of kin. Written informed consent was obtained from the individual(s) for the publication of any potentially identifiable images or data included in this article.

## Author contributions

QF: Writing – original draft, Writing – review & editing. MD: Data curation, Writing – original draft. WC: Data curation, Writing – review & editing. LS: Data curation, Writing – review & editing. YZ: Writing – review & editing. QL: Formal analysis, Writing – review & editing. ZW: Writing – original draft, Writing – review & editing.

## Supplementary material

SUPPLEMENTARY FIGURE S1 Self-made teaching pictures for the song "Hello".

SUPPLEMENTARY FIGURE S2 Orff Music Therapy Class Photo: imitating Animal Voices, introducing "Harvest Song" in the dance section, singing songs such as "Hello", "Making a Call", and "I Can Cross the Street", and using the music instrument of a bell drum in the song "I Can Cross the Street".
