# Supplementary figures and images for: The clinical effects of Orff music therapy on children with autism spectrum disorder: a comprehensive evaluation
Source: Front Neurol. 2024 Jun 6;15:1387060. doi: 10.3389/fneur.2024.1387060 (PMC11188925; doi:10.3389/fneur.2024.1387060)

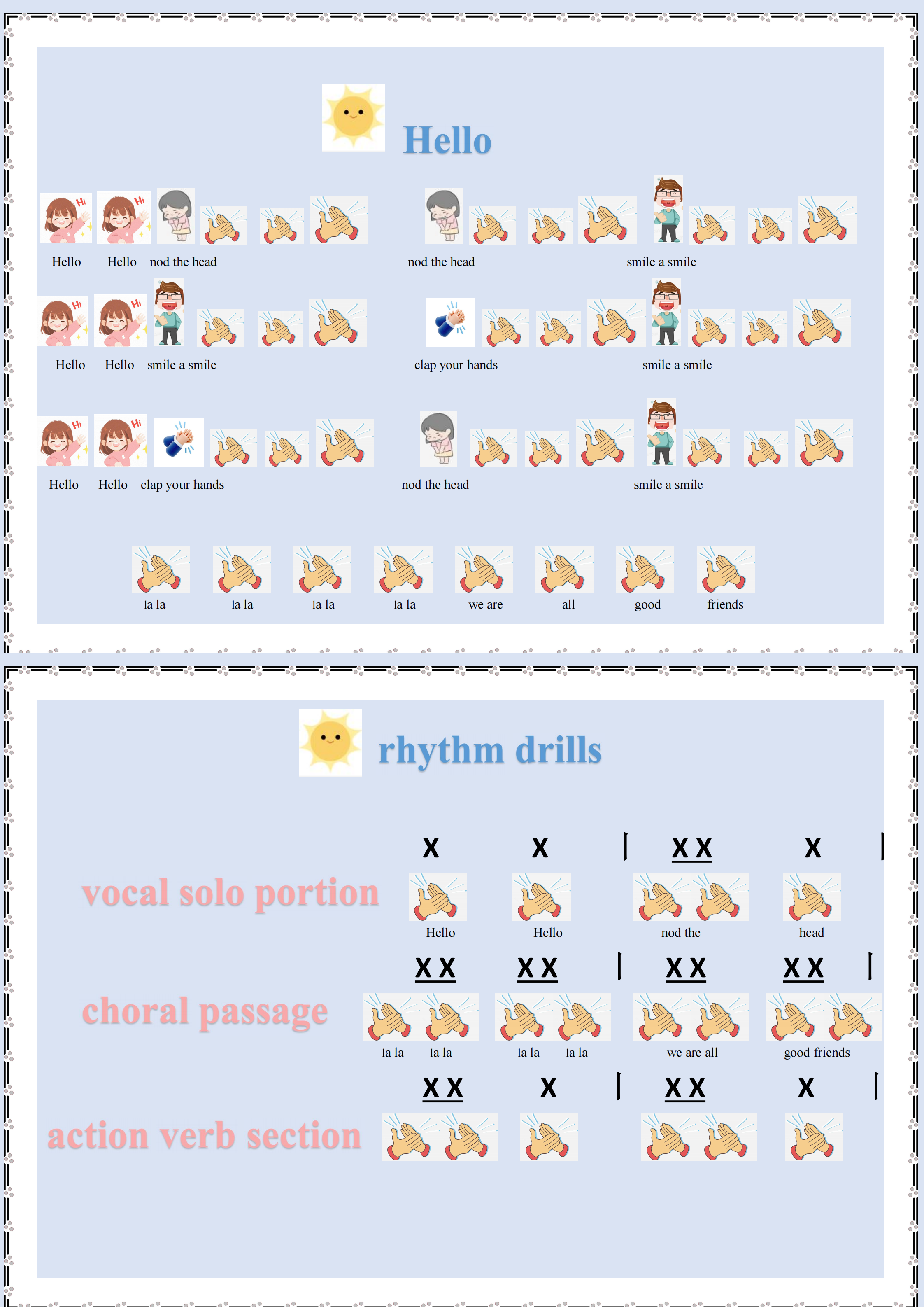

Supplement: SUPPLEMENTARY FIGURE S1 — Self-made teaching pictures for the song "Hello". [file Image_1.TIF]

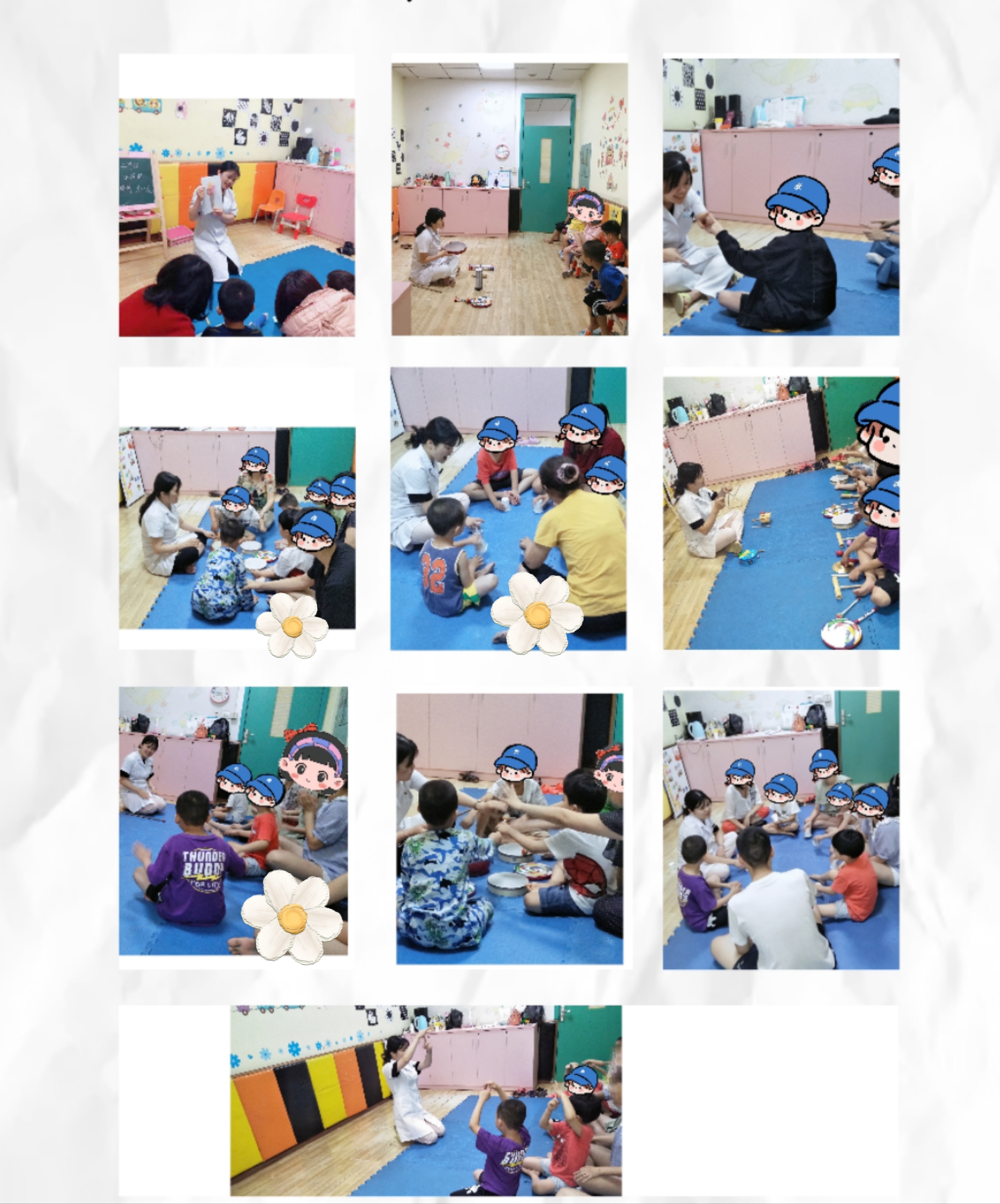

Supplement: SUPPLEMENTARY FIGURE S2 — Orff Music Therapy Class Photo: imitating Animal Voices, introducing "Harvest Song" in the dance section, singing songs such as "Hello", "Making a Call", and "I Can Cross the Street", and using the music instrument of a bell drum in the song "I Can Cross the Street". [file Image_2.JPEG]
