# Supplementary material for: The clinical effects of Orff music therapy on children with autism spectrum disorder: a comprehensive evaluation
Source: Front Neurol. 2024 Jun 6;15:1387060. doi: 10.3389/fneur.2024.1387060 (PMC11188925; doi:10.3389/fneur.2024.1387060)
Supplement: Supplementary file 7 [file Data_Sheet_5.DOCX]

| **Orff Music Therapy Course A** | | | | | | | | | | |
| --- | --- | --- | --- | --- | --- | --- | --- | --- | --- | --- |
| **Teaching curriculum** | **Song: "Hello"** | | **Therapist** | | Mengying Ding | | **Class schedule** | | November 9th, 2023, 15:20- 16:00 | |
| **Individualized teaching objectives** | **Course objective** | | Participant's name | | | | | | | |
|  |  |  | Child A | | Child B | | Child C | | Child D | Child E |
|  | 1. Familiarity with lyrics,   guiding language stimuli, familiarity with "greeting" expressions during introductions. | | √ | | √ | | √ | | √ | √ |
|  | **2**.Promoting participation in two-player or parent-child games not only fosters self-confidence and facilitates parent-child interaction, but also enhances the development of psychomotor skills. | | √ | | √ | | √ | | √ | √ |
|  | **3**.The rhythmic tapping of a four-two beat can be imitated, either through body movements or by using musical instruments, to promote rhythm perception and coordination. | | √ | | √ | | √ | | √ | √ |
|  | **4.**Fostering rhythm perception and developing a sense of rhythm can contribute to the improvement of body balance through sensory stimulation. | | √ | | √ | | √ | | √ | √ |
| **Teaching objectives and challenges** | **1.**Children with ASD can independently initiate greetings and experience joy in the process.  **2.**Children with ASD demonstrate the ability to maintain attention, exhibit imitation of actions, and show a desire to actively engage in music activities. | | | | | | | | | |
| **Session One** | | | | | | | | | | |
| **Materials Preparation** | 1. Audio file of the song "Hello" 2. Self-made teaching pictures for the song "Hello" (Figure S1) . 3. Hand drum, tambourine | | | | | | | | | |
| **Teaching process** (Figure S2) | **Segment One (5min):** Introduction through A Cappella Greeting   1. The instructor, seated on a cushion, greets everyone. 2. The instructor demonstrates the rhythm of greeting by clapping hands and guides the parents to respond with clapping. The clapping rhythm is as follows：   4/4 X X X— \| X X X—  \|\|  Children A Hello  Hello dear teacher   1. The teacher guides all parents to greet the child in a rhythmic manner using the same method. | | | | | | | | | |
|  | **Segment Two (25min):** Dialogue Introduction  Children, how do you react when you meet a good friend or someone familiar? (Parents guide children to answer the question)   1. Listen to the audio of the song "Hello" for the first time, the teacher demonstrates actions, and parents guide children to pay attention to the actions representing the lyrics. 2. During the second listening of the song "Hello", the teacher will show self-made teaching images and guide students to follow the rhythm and melody, while demonstrating the clapping movements. 3. During the third listening of the song "Hello," instructors guide parents and children to follow self-made teaching images and participate in various forms of group activities, including duet, parent-child, and instrument-based performances. | | | | | | | | | |
| **Further Extension of the Curriculum（10min）** | During the third playback of the audio for the song "Hello," all participating children with ASD engaged in physical rhythmic movements and tapped their hands to the beat. Upon hearing the prompts "nod your head," "smile," "clap your hands," "bow," "pat shoulders," and "shake hands," the children took turns demonstrating these actions, while those not demonstrating continued to tap the rhythm. Subsequently, the music was played again, and the parents and children linked hands to form a circle, stepping in time with the rhythm of the song. Upon hearing actions such as "nod your head," the parents and children demonstrated these actions to each other. Ultimately, the children were able to use various instruments and scenarios to simulate the scenario of meeting and saying "Hello". | | | | | | | | | |
| **Reflections on the Curriculum** | Although the content of this lesson was straightforward, some children with ASD exhibited impaired cognitive functions, which may have compromised the efficacy of this session for them. These children often appeared to merely mimic the instructions. Conversely, those with relatively intact cognition demonstrated higher completion rates during the initial part of the course. However, as the lesson progressed, the reduced ability of ASD children to maintain focused attention and a decline in their engagement with the material to a decrease in the efficiency of the latter part of the session. Post-lesson, it is imperative for educators to address these issues by refining the instructional plan. Strategies may include providing pre-lesson motor previews for children with lower cognitive abilities and implementing post-lesson reviews to reinforce learning. Additionally, more advanced goals tailored to the capabilities of children with relatively higher cognitive functioning could be established. | | | | | | | | | |
| **Achievement of Objectives** | | | | | | | | | | |
| **Child A** | | | | | | | | | | |
|  | Course Objectives | Teaching Strategies | | Implementation Status | | Teaching Decisions | | Therapist | | |
| **Course completion status** | 1.Establishing Classroom Rules(long-term). | V | | 4 | | P | | Mengying Ding | | |
|  | 1.1 Emotional Regulation. | V | | 4 | | P | | Mengying Ding | | |
|  | 1.2 Sitting ability. | V | | 4 | | P | | Mengying Ding | | |
|  | 2.Imitation of Actions (Short-Term). | V | | 4 | | P | | Mengying Ding | | |
|  | 3.Body rhythm, rhythmic tapping. | V | | 3 | | P | | Mengying Ding | | |
|  | 4.Singing. | V | | 3 | | P | | Mengying Ding | | |
| **Conclusion:** Child A acquired the skill of handshaking and brief eye contact with others. However, they continue to exhibit difficulty in accepting physical contact during play activities. | | | | | | | | | | |
| **Child B** | | | | | | | | | | |
|  | Course Objectives | Teaching Strategies | | Implementation Status | | Teaching Decisions | | Therapist | | |
| **Course completion status** | 1.Establishing Classroom Rules.(long-term) | V | | 4 | | P | | Mengying Ding | | |
|  | 1.1 Emotional Regulation. | V | | 4 | | P | | Mengying Ding | | |
|  | 1.2 Sitting ability. | V | | 4 | | P | | Mengying Ding | | |
|  | 2.Imitation of Actions (Short-Term). | M | | 3 | | P | | Mengying Ding | | |
|  | 3.Body rhythm, rhythmic tapping. | M | | 3 | | P | | Mengying Ding | | |
|  | 4.Singing. | P1 | | 3 | | P | | Mengying Ding | | |
| **Conclusion:** Child B demonstrated significant progress in imitating actions during this class session, but lacked confidence in imitating sounds, requiring substantial assistance. | | | | | | | | | | |
| **Child C** | | | | | | | | | | |
|  | Course Objectives | Teaching Strategies | | Implementation Status | | Teaching Decisions | | Therapist | | |
| **Course completion status** | 1.Establishing Classroom Rules.(long-term) | V | | 4 | | P | | Mengying Ding | | |
|  | 1.1 Emotional Regulation. | V | | 4 | | P | | Mengying Ding | | |
|  | 1.2 Sitting ability. | V | | 4 | | P | | Mengying Ding | | |
|  | 2.Imitation of Actions (Short-Term). | V | | 4 | | P | | Mengying Ding | | |
|  | 3.Body rhythm, rhythmic tapping. | V | | 4 | | P | | Mengying Ding | | |
|  | 4.Singing. | V | | 4 | | P | | Mengying Ding | | |
| **Conclusion:** Child C demonstrated good compliance with classroom rules and was able to follow teacher instructions to complete corresponding movements, and showed a moderate ability to imitate and follow rhythmic patterns, although creativity and originality still need further enhancement. | | | | | | | | | | |
| **Child D** | | | | | | | | | | |
|  | Course Objectives | Teaching Strategies | | Implementation Status | | Teaching Decisions | | Therapist | | |
| **Course completion status** | 1.Establishing Classroom Rules.(long-term) | V | | 4 | | P | | Mengying Ding | | |
|  | 1.1 Emotional Regulation. | V | | 4 | | P | | Mengying Ding | | |
|  | 1.2 Sitting ability. | V | | 4 | | P | | Mengying Ding | | |
|  | 2.Imitation of Actions (Short-Term). | M | | 3 | | P | | Mengying Ding | | |
|  | 3.Body rhythm, rhythmic tapping. | M | | 2 | | P | | Mengying Ding | | |
|  | 4.Singing. | M | | 3 | | P | | Mengying Ding | | |
| **Conclusion:** Child D demonstrated good compliance with classroom rules; however, they exhibited limited initiative in imitating actions, sounds, and greetings. Additionally, they require assistance to improve their coordination skills. | | | | | | | | | | |
| **Child E** | | | | | | | | | | |
|  | Course Objectives | Teaching Strategies | | Implementation Status | | Teaching Decisions | | Therapist | | |
| **Course completion status** | 1.Establishing Classroom Rules.(long-term) | V | | 4 | | P | | Mengying Ding | | |
|  | 1.1 Emotional Regulation. | V | | 4 | | P | | Mengying Ding | | |
|  | 1.2 Sitting ability. | V | | 4 | | P | | Mengying Ding | | |
|  | 2.Imitation of Actions (Short-Term). | M | | 3 | | P | | Mengying Ding | | |
|  | 3.Body rhythm, rhythmic tapping. | M | | 2 | | P | | Mengying Ding | | |
|  | 4.Singing. | M | | 2 | | P | | Mengying Ding | | |
| **Conclusion:** Child E demonstrated good compliance with classroom rules and had relatively strong social and language abilities, but lacked initiative. It is necessary to build confidence and self-esteem in later classrooms to enhance their participation and engagement. | | | | | | | | | | |

**Teaching Strategies：**P1: Large Amount of Assistance; P2: Small Amount of Assistance； M: Modeling and Teaching；V: Verbal Tips；G: Body Assistance.

**Implementation Status：**0: Less than 25%；1:Achieved 25%；2: Achieved 50%; 3: Achieved 75%; 4: Achieved 100%.

**Teaching Decisions：**P: Continuing; S: Replacement.
