# Supplementary material for: The clinical effects of Orff music therapy on children with autism spectrum disorder: a comprehensive evaluation
Source: Front Neurol. 2024 Jun 6;15:1387060. doi: 10.3389/fneur.2024.1387060 (PMC11188925; doi:10.3389/fneur.2024.1387060)
Supplement: Supplementary file 8 [file Data_Sheet_6.DOCX]

| **Orff Music Therapy Course B** | | | | | | | | | | |
| --- | --- | --- | --- | --- | --- | --- | --- | --- | --- | --- |
| **Teaching curriculum** | **Song : "Hello"** | | **Therapist** | | Mengying Ding | | **Class schedule** | | November 13th, 2023, 15:20- 16:00 | |
| **Individualized teaching objectives** | **Course objective** | | Participant's name | | | | | | | |
|  |  |  | Child A | | Child B | | Child C | | Child D | Child E |
|  | 1. Enhancing language and cognitive abilities through imitation of animal movements and sounds. | | √ | | √ | | √ | | √ | √ |
|  | 1. Engaging in peer communication and interaction under the guidance of therapists and parents, demonstrating flexibility in using waiting, taking turns, and collaborating. | | √ | | √ | | √ | | √ | √ |
|  | **3**.Training for name recognition, greeting treatment rooms upon arrival and departure, teaching social etiquette, enhancing attention to peers, and improving responsiveness to group instructions from the therapist. | | √ | | √ | | √ | | √ | √ |
|  | 1. Children A and C were able to move to music, learn song lyrics, and complete actions with minimal assistance, whereas children B, D, and E required semi-supported guidance to accomplish the movements. | | √ | | √ | | √ | | √ | √ |
| **Teaching objectives and challenges** | 1.Developing auditory abilities and music perception in children with ASD.  2.Improving physical coordination and focus in children with ASD. | | | | | | | | | |
| **Session One** | | | | | | | | | | |
| **Materials Preparation** | 1. Audio file of the song "Hello" 2. Self-made teaching pictures for the song "Hello" (Figure S1). 3. Pictures of kittens, puppies, and bees. 4. Shaker | | | | | | | | | |
| **Teaching process** (Figure S2) | **Segment one (5min):**  The therapist demonstrates how to perform a rhythmic greeting with two syllables per beat.  \|XX    XX   XX   X\|\|  Children A   Children B Good morning  Parents are instructed to facilitate hand-clapping responses from the children.  \|XX    XX     XX   X\|\|  Teacher  Teacher Good morning  The therapist initiates a rhythmic hand-clapping greeting with the children one by one, while the parents assist their children in responding to the therapist with hand-clapping.  The therapist plays the song "Hello" to welcome all the children and parents, and to review the content of the previous session, recalling activities that involved greeting others in various ways. | | | | | | | | | |
|  | **Segment two (25min)**:  Presenting pictures of kittens, puppies, and bees, listening to their corresponding animal sounds, and imitating those sounds and movements, greeting each other with animal noises and actions.  **At session one:** Hello, hello, clap your hands.  **Now, in session two:** Hello, hello, meow, meow, meow or Hello, hello, woof, woof, woof.  **Engaging in collaborative participation:** "Meow, Woof, Buzz" and creating movements together with parents.  **Rhythmic game:** Walking together in a circle, gathering and dispersing upon hearing animal sounds.  **Instrument Playing:** Shaker Demonstration: For the puppy, place the shaker on their head; for the kitten, place the shaker next to their mouth, and for the bee, place the shaker in their hand and shake it.  Children with lower abilities can simplify the movements by performing them in place or focusing on only one element. Children with higher abilities can increase the complexity by engaging in improvised role-play. For example, "Kittens meet kittens, greeting with meows, meows. Dogs meet dogs, greeting with woofs, woofs. Bees find bees, fluttering wings to greet each other." This can be practiced using a combination of rap, movements, and musical instruments. | | | | | | | | | |
| **Further Extension of the Curriculum（10min）** | Having become familiar with the music, children may be permitted to select their own instruments. Subsequently, they can use these instruments to perform in accordance with the music. Ultimately, they should be able to simulate meeting scenarios and greet each other with phrases like "Hello" using a variety of instruments and different contexts. | | | | | | | | | |
| **Reflections on the Curriculum** | In comparison to the previous lesson, this lesson has expanded the use of animal imitations such as "meow meow meow" and "woof woof woof" instead of the traditional actions such as "clapping hands" and "nodding heads" in the phrase "hello hello clapping hands". Overall, children with ASD showed better completion and higher levels of collaboration and creativity. However, there are areas for improvement: 1. The lesson structure should be more clear and detailed. 2. In the classroom teaching process, the pace should be slowed down for children with different abilities, and more exaggerated gestures and body movements should be used to guide them. | | | | | | | | | |
| **Achievement of Objectives** | | | | | | | | | | |
| **Child A** | | | | | | | | | | |
|  | Course Objectives | Teaching Strategies | | Implementation Status | | Teaching Decisions | | Therapist | | |
| **Course completion status** | 1.Establishing Classroom Rules.(long-term) | V | | 4 | | P | | Mengying Ding | | |
|  | 1.1 Emotional Regulation. | V | | 4 | | P | | Mengying Ding | | |
|  | 1.2 Sitting ability. | V | | 4 | | P | | Mengying Ding | | |
|  | 2.Able to follow music and teacher's movements to imitate animal actions. | V | | 4 | | P | | Mengying Ding | | |
|  | 3.Imitating animal sounds. | V | | 3 | | P | | Mengying Ding | | |
|  | 4.Singing. | V | | 3 | | P | | Mengying Ding | | |
|  | 5.At the end, able to assist the teacher in tidying up teaching materials. | V | | 4 | | P | | Mengying Ding | | |
| **Conclusion:** Child A was able to follow and complete movements in this lesson, demonstrating independent body transitions accompanied by music. | | | | | | | | | | |
| **Child B** | | | | | | | | | | |
|  | Course Objectives | Teaching Strategies | | Implementation Status | | Teaching Decisions | | Therapist | | |
| **Course completion status** | 1.Establishing Classroom Rules.(long-term) | V | | 4 | | P | | Mengying Ding | | |
|  | 1.1 Emotional Regulation. | V | | 4 | | P | | Mengying Ding | | |
|  | 1.2 Sitting ability. | V | | 4 | | P | | Mengying Ding | | |
|  | 2.Able to follow music and teacher's movements to imitate animal actions. | M | | 3 | | P | | Mengying Ding | | |
|  | 3.Imitating animal sounds. | P2 | | 3 | | P | | Mengying Ding | | |
|  | 4.Singing. | P1 | | 3 | | P | | Mengying Ding | | |
|  | 5.At the end, able to assist the teacher in tidying up teaching materials. | M | | 2 | | P | | Mengying Ding | | |
| **Conclusion:** Child B demonstrated relatively stable emotions and overall good cooperation throughout the lesson, with intermittent participation in singing, although additional verbal reminders were required. | | | | | | | | | | |
| **Child C** | | | | | | | | | | |
|  | Course Objectives | Teaching Strategies | | Implementation Status | | Teaching Decisions | | Therapist | | |
| **Course completion status** | 1.Establishing Classroom Rules.(long-term) | V | | 4 | | P | | Mengying Ding | | |
|  | 1.1 Emotional Regulation. | V | | 4 | | P | | Mengying Ding | | |
|  | 1.2 Sitting ability. | V | | 4 | | P | | Mengying Ding | | |
|  | 2.Able to follow music and teacher's movements to imitate animal actions. | M | | 3 | | P | | Mengying Ding | | |
|  | 3.Imitating animal sounds. | V | | 3 | | P | | Mengying Ding | | |
|  | 4.Singing. | V | | 3 | | P | | Mengying Ding | | |
|  | 5.At the end, able to assist the teacher in tidying up teaching materials. | V | | 3 | | P | | Mengying Ding | | |
| **Conclusion:** Child C demonstrated familiarity with music melodies and was able to independently perform various body movements under imitation conditions. They also showed the ability to praise others by saying "You're great" at the conclusion of their performance. | | | | | | | | | | |
| **Child D** | | | | | | | | | | |
|  | Course Objectives | Teaching Strategies | | Implementation Status | | Teaching Decisions | | Therapist | | |
| **Course completion status** | 1.Establishing Classroom Rules.(long-term) | V | | 4 | | P | | Mengying Ding | | |
|  | 1.1 Emotional Regulation. | V | | 4 | | P | | Mengying Ding | | |
|  | 1.2 Sitting ability. | V | | 4 | | P | | Mengying Ding | | |
|  | 2.Able to follow music and teacher's movements to imitate animal actions. | P2 | | 3 | | P | | Mengying Ding | | |
|  | 3.Imitating animal sounds. | P2 | | 2 | | P | | Mengying Ding | | |
|  | 4.Singing. | P2 | | 3 | | P | | Mengying Ding | | |
|  | 5.At the end, able to assist the teacher in tidying up teaching materials. | G | | 2 | | P | | Mengying Ding | | |
| **Conclusion:** Child D was able to intermittently join in singing and completedsong movements with semi-assistance. | | | | | | | | | | |
| **Child E** | | | | | | | | | | |
|  | Course Objectives | Teaching Strategies | | Implementation Status | | Teaching Decisions | | Therapist | | |
| **Course completion status** | 1.Establishing Classroom Rules.(long-term) | V | | 4 | | P | | Mengying Ding | | |
|  | 1.1 Emotional Regulation. | V | | 4 | | P | | Mengying Ding | | |
|  | 1.Establishing Classroom Rules.(long-term) | V | | 4 | | P | | Mengying Ding | | |
|  | 1.1 Emotional Regulation. | V | | 4 | | P | | Mengying Ding | | |
|  | 1.2 Sitting ability. | V | | 4 | | P | | Mengying Ding | | |
|  | 2.Able to follow music and teacher's movements to imitate animal actions. | G | | 3 | | P | | Mengying Ding | | |
|  | 3.Imitating animal sounds. | P2 | | 2 | | P | | Mengying Ding | | |
| **Conclusion:** Child E exhibited intermittent singing along with the songs but required supervision for improved cooperation, and completed imitative movements with minimal assistance. | | | | | | | | | | |

**Teaching Strategies：**P1: Large Amount of Assistance; P2: Small Amount of Assistance； M: Modeling and Teaching；V: Verbal Tips；G: Body Assistance.

**Implementation Status：**0: Less than 25%；1:Achieved 25%；2: Achieved 50%; 3: Achieved 75%; 4: Achieved 100%.

**Teaching Decisions：**P: Continuing; S: Replacement.
