# Supplementary material for: The clinical effects of Orff music therapy on children with autism spectrum disorder: a comprehensive evaluation
Source: Front Neurol. 2024 Jun 6;15:1387060. doi: 10.3389/fneur.2024.1387060 (PMC11188925; doi:10.3389/fneur.2024.1387060)
Supplement: Supplementary file 9 [file Table_1.docx]

| **TABLE** **S1** Summary of music therapy for autism spectrum disorder in children | | | | | | |
| --- | --- | --- | --- | --- | --- | --- |
| Study | Sample Size | Age | Intervention | | Intervention time | Outcome |
|  |  |  | Observation group | Control group |  |  |
| LaGasse 2014(1) | Total=17  Boys=13  Girls=4 | 6-9 years | n=9  MT | n=8  social skills | 5 weeks | Global improvement  Social interaction  Non-verbal communication  Total autism symptom severity |
| Ghasemtabar 2015(2) | Total=27  Boys=14  Girls=13 | 7-12 years | n=13  MT Orff-Schulwerk | n=14  standard care | 45 days | Social interaction |
| Huang 2015(3) | Total=60  Boys=47  Girls=13 | 2-9 years | n=30  integrated therapy plus MT | n=30  integrated therapy | 2 months | Total autism symptom severity |
| Porter 2017(4) | Total=47  Boys=34  Girls=13 | 8-16 years | n=24  MT | n=23  TAU | 12 weeks | Global improvement  Social interaction  Adverse events  Adaptive behaviour  Quality of family relationships  Identity formation  Depression |
| Bieleninik 2017(5) | Total=364  Boys=302  Girls=62 | 4-6.11 years | n=182  individual improvisational music therapy | n=182  standard care | 5 months | Global improvement  Social interaction  Quality of life  Total autism symptom severity  Adverse events  Adaptive behaviour |
| Sharda 2018(6) | Total=51  Boys=43  Girls=8 | 6-12 years | n=26  MT | n=25  play-based intervention | 8-12 weeks | Social interaction  Non-verbal communication  Verbal communication  Quality of life  Adaptive behaviour |
| Bharathi 2019(7) | Total=52  Boys=26  Girls=26 | 6-12 years | n=26  MT | n=26  music listening | 3 months | Global improvement  Social interaction  Total autism symptom severity |
| Rabeyron 2020(8) | Total=37  Boys=32  Girls=5 | 4-7 years | n=19  MT | n=18  music listening | 8 months | Global improvement  Social interaction  Non-verbal communication  Verbal communication  Total autism symptom severity  Adaptive behaviour |

MT: music therapy; TAU: treatment-as-usual

**References:**

1.LaGasse AB. Effects of a music therapy group intervention on enhancing social skills in children with autism. *J. Music Ther.*(2014) 51(3): 250-75. doi:10.1093/jmt/thu012

2.Ghasemtabar SN, Hosseini M, Fayyaz I, Arab S, Naghashian H, Poudineh Z. Music therapy: An effective approach in improving social skills of children with autism. *Adv. Biomed. Res.*(2015) 4(157. doi:10.4103/2277-9175.161584

3.Huang HC, Wang LH, Chang HJ. [Mental Health Promotion Among the Chronic Disabled Population in the Community]. *Hu Li Za Zhi*.(2015) 62(4): 34-40. doi:10.6224/JN62.4.34

4.Porter S, McConnell T, McLaughlin K, Lynn F, Cardwell C, Braiden HJ, et al. Music therapy for children and adolescents with behavioural and emotional problems: a randomised controlled trial. *J. Child Psychol. Psychiatry*.(2017) 58(5): 586-94. doi:10.1111/jcpp.12656

5.Bieleninik L, Geretsegger M, Mossler K, Assmus J, Thompson G, Gattino G, et al. Effects of Improvisational Music Therapy vs Enhanced Standard Care on Symptom Severity Among Children With Autism Spectrum Disorder: The TIME-A Randomized Clinical Trial. *JAMA-J. Am. Med. Assoc.*(2017) 318(6): 525-35. doi:10.1001/jama.2017.9478

6.Sharda M, Tuerk C, Chowdhury R, Jamey K, Foster N, Custo-Blanch M, et al. Music improves social communication and auditory-motor connectivity in children with autism. *Transl. Psychiatr.*(2018) 8(1): 231. doi:10.1038/s41398-018-0287-3

7.Bharathi G, Jayaramayya K, Balasubramanian V, Vellingiri B. The potential role of rhythmic entrainment and music therapy intervention for individuals with autism spectrum disorders. *J. Exerc. Rehabil.*(2019) 15(2): 180-6. doi:10.12965/jer.1836578.289

8.Rabeyron T, Robledo DCJ, Carasco E, Bisson V, Bodeau N, Vrait FX, et al. A randomized controlled trial of 25 sessions comparing music therapy and music listening for children with autism spectrum disorder. *Psychiatry Res.*(2020) 293(113377. doi:10.1016/j.psychres.2020.113377
